# Differentiation-Dependent Motility-Responses of Developing Neural Progenitors to Optogenetic Stimulation

**DOI:** 10.3389/fncel.2017.00401

**Published:** 2017-12-19

**Authors:** Tímea Köhidi, Attila G. Jády, Károly Markó, Noémi Papp, Tibor Andrási, Zsuzsanna Környei, Emília Madarász

**Affiliations:** ^1^Laboratory of Cellular and Developmental Neurobiology, Institute of Experimental Medicine, Hungarian Academy of Sciences, Budapest, Hungary; ^2^Roska Tamás Doctoral School of Sciences and Technology, Faculty of Information Technology and Bionics, Pázmány Péter Catholic University, Budapest, Hungary; ^3^Adult Stem Cell Section, National Institute of Dental and Craniofacial Research, National Institutes of Health, Bethesda, MD, United States; ^4^Lendület Laboratory of Network Neurophysiology, Institute of Experimental Medicine, Hungarian Academy of Sciences, Budapest, Hungary; ^5^Laboratory of Neuroimmunology, Institute of Experimental Medicine, Hungarian Academy of Sciences, Budapest, Hungary

**Keywords:** radialglia-like stem cells, *in vitro* neurogenesis, cell motility, optogenetic stimulation

## Abstract

During neural tissue genesis, neural stem/progenitor cells are exposed to bioelectric stimuli well before synaptogenesis and neural circuit formation. Fluctuations in the electrochemical potential in the vicinity of developing cells influence the genesis, migration and maturation of neuronal precursors. The complexity of the *in vivo* environment and the coexistence of various progenitor populations hinder the understanding of the significance of ionic/bioelectric stimuli in the early phases of neuronal differentiation. Using optogenetic stimulation, we investigated the *in vitro* motility responses of radial glia-like neural stem/progenitor populations to ionic stimuli. Radial glia-like neural stem cells were isolated from *CAG^loxp^Stop^loxp^ChR2(H134)-eYFP* transgenic mouse embryos. After transfection with Cre-recombinase, ChR2(channelrhodopsin-2)-expressing and non-expressing cells were separated by eYFP fluorescence. Expression of light-gated ion channels were checked by patch clamp and fluorescence intensity assays. Neurogenesis by ChR2-expressing and non-expressing cells was induced by withdrawal of EGF from the medium. Cells in different (stem cell, migrating progenitor and maturing precursor) stages of development were illuminated with laser light (λ = 488 nm; 1.3 mW/mm^2^; 300 ms) in every 5 min for 12 h. The displacement of the cells was analyzed on images taken at the end of each light pulse. Results demonstrated that the migratory activity decreased with the advancement of neuronal differentiation regardless of stimulation. Light-sensitive cells, however, responded on a differentiation-dependent way. In non-differentiated ChR2-expressing stem cell populations, the motility did not change significantly in response to light-stimulation. The displacement activity of migrating progenitors was enhanced, while the motility of differentiating neuronal precursors was markedly reduced by illumination.

## Introduction

Developing neural cells are exposed to depolarizing agents in the entire period of neuronal differentiation, from cell generation and migration up to the circuit integration of newly generated neurons. Depolarization, by modifying the space and time distribution of intracellular ions, can regulate basic cell physiological processes.

Depolarizing stimuli affect early neural progenitors *via* multiple routes including ion fluxes through voltage-dependent or ligand-gated ion channels (Jelitai et al., 2004, 2007) and Ca-release from IP_3_-sensitive Ca-stores (Bolteus and Bordey, 2004). The expression of ligand-gated and voltage-sensitive ion channels changes with the advancement of neuronal differentiation (LoTurco et al., 1995; Jelitai et al., 2007), consequently, the response of neural stem/progenitor cells to depolarizing stimuli will depend on the actual stage of cell development and also on the characteristics of the affected cells. In proliferating cells, membrane depolarization can regulate the progression through the cell cycle *via* altered intracellular Ca ^⋅^([Ca^2+^]_IC_) oscillations (Jacobson, 1978; Herberth et al., 2002; Weissman et al., 2004). In migrating progenitors, cell displacement, e.g., the formation of leading lamellipodia and generation of contractile forces are sensitively regulated by the level of intracellular free Ca^2+^. Changes in the free intracellular Ca^2+^ pool can modulate the outgrowth, elongation and pathfinding of neurites of differentiating neuronal precursors (Gomez et al., 2001; Henley and Poo, 2004).

Intracellular ion responses can be initiated by multiple extracellular stimuli including receptor mediated actions of growth factors and neurotransmitters (Ge et al., 2006; Flavell and Greenberg, 2008; Song et al., 2012), direct depolarizing effects of spreading bioelectric signals (O’Donovan, 1999) and shifts in the ion composition of the extracellular fluid.

The environment of stem, progenitor or neuronal precursor cells enclose all of these agents: it contains neurotransmitters and growth factors, displays important ion fluctuations and mediates spreading bioelectric fluctuations (Ge et al., 2006; Spitzer, 2006; Flavell and Greenberg, 2008; Song et al., 2012; Suárez et al., 2014; Luhmann et al., 2016). Neural stem/progenitor cells are depolarized by GABA which is known to be an important constituent of the neural tissue environment in all stages of development (Benítez-Diaz et al., 2003; Jelitai and Madarasz, 2005; Song et al., 2012). Spontaneous Ca-oscillations are spreading through gap junctions in the early neural tube (O’Donovan, 1999), and giant depolarizing potentials are traveling along the growing neurites in the developing brain (Ben-Ari, 2001) before and during the formation of synaptically coupled neuronal networks. External stimuli-caused potential changes influence the migration and integration of neuronal precursors in the adult hippocampus, as well (Parent et al., 1997; Ge et al., 2006; Song et al., 2012).

In the developing central nervous system, multiple types and developmental stages of neural stem/progenitor cells coexist (Madarász, 2013). The time- and space-coordinated migration of neural progenitors is a basic phenomenon of the neural tissue genesis (Rakic, 1971; Kriegstein and Noctor, 2004). The delicate spatial-temporal maps of the migratory routes are outlined by the different expression of cell adhesion molecules, by the composition of deposited extracellular matrix (ECM) components and by the differences in surface receptors carried by subpopulations of cells. Depolarizing agents contribute to patterning the migration, mainly through transient modifications of the local [Ca^2+^]_IC,_ which is known to regulate cell contractility, deposition of ECM, secretion of growth factors and the release of GABA (Madarász, 2013).

The complexity of the *in vivo* environment and the coexistence of various progenitor populations hinder the understanding of the significance of ionic/bioelectric stimuli in the early phases of neuronal differentiation. We conducted *in vitro* motility studies on well-characterized radial glia-like (RGl) neural stem/progenitor cells (Markó et al., 2011) (see Supplementary Material S1) in order to explore the migratory responses of RGl cells in different stages of *in vitro* neuronal differentiation.

Populations of RGl cells can be prepared from the mouse brain at various ages including embryos and aged adult animals (Markó et al., 2011; Madarász, 2013) (see also Supplementary Material S1). RGl cells, if adhered to an artificial peptide (AK-cyclo[RGDfC]) composed by short alanine *n* = 3–5 side chains conjugated to every 4th to 8th lysine in long poly-lysine chains and carrying cyclic RGD motifs at the free end; Markó et al., 2008) survive selectively in serum-free medium in the presence of EGF and insulin as only growth factor supplements. The cells display elongated epithel-like morphology, glial fibrillary acidic protein (GFAP), RC2 and nestin immunoreactivity, express the *pax6* (paired box 6), *sox2* (sex determining region Y-box 2), and *blbp* (brain lipid binding protein coding) radial glia marker genes and proliferate with an approximately 24-h duplication time. Upon withdrawal of EGF, a number of cells die, while about 20% of cells acquire neuronal characteristics by the 5th to 7th day of EGF withdrawal (Markó et al., 2011) (see also Supplementary Material S1).

For studying the cation influx elicited motility responses, radial glia-like (RGl) neural stem cells were isolated from channelrhodopsin-2 (ChR2) gene carrying transgenic mouse embryos, and illumination was used for non-invasive stimulation (Zhang et al., 2010). The *in vitro* neurogenesis of optogenetically modified RGl cells allowed investigating the effects of non-invasive ionic stimulation on the displacement activity of RGl stem cells, RGl-derived progenitors and maturing neuronal precursors.

## Materials and Methods

### Isolation of Radial Glia-Like Neural Stem Cells from the Forebrain of B6;129S-Gt(ROSA)26Sor ^tm32(CAG-COP4^∗^H134R/EY FP)Hze ^/J Transgenic Mouse Fetuses

The isolation procedure was done according to the animal experimentation license 22.1/354/3/2011 (issued by the National Food-chain Safety Office) and was executed as described before (Markó et al., 2011). Briefly, timed pregnant B6;129S-Gt(ROSA)26Sor^tm32(CAG-COP4^∗^H134R/EY FP)Hze^/J mice (Madisen et al., 2012) were sacrificed on 17.5 post-conception day by intraperitoneal injection of 100 μg/g ketamine (CP-Pharma mbH, Germany) and 10 μg/g xylazine (CEVA-Phylaxia, Hungary). Brains of the embryos were aseptically removed, telencephali were isolated and placed into sterile phosphate buffered saline (PBS). Under stereomicroscope the meninges were removed and the tissue was manually cut into small pieces (∼1 mm^3^). The pieces were suspended in DMEM (Sigma) and triturated with Pasteur-pipette. The cell suspension was filtered through a nylon mesh with pore diameter of 45 μm. The cells were sedimented by centrifugation (120 g; 10 min) and the pellet was quickly re-suspended in DMEM/F12 (1/1) medium (Sigma) containing 1% B27 (Gibco). The cells (6 × 10^6^ cells/cm^2^) were plated into 60 mm Petri-dishes (Falcon) coated with a synthetic AK-cyclo[RGDfC] peptide (Markó et al., 2008) at 0.25 μg/cm^2^ surface density. The culture medium was supplemented with EGF (20 ng/ml; Peprotech), as the only exogenous growth factor in addition to insulin present in B27. The culture medium was changed every second day and the cells were harvested by trypsinization when reached confluency. After 3–4 passages, cultures were composed by virtually homogeneous populations of proliferating radial glia-like cells (Markó et al., 2011). The radial glial nature of cultivated cells was checked by PCR studies on gene expression according to Markó et al. (2011) and by immunocytochemical investigations of cell-type specific markers (see Supplementary Material S1).

### Transfection with pTurbo-Cre Plasmid

Radial glia-like cells containing *loxpStoploxpChR2(H134)-EYFP* construct were grown in 4-well plates (5 × 10^4^ cells/well) and were transfected with 1 μg pTurbo-Cre plasmid [Cre expression driven by chicken-β-actin promoter (Hug et al., 1996)] using Lipofectamin-Plus reagent (Invitrogen-Thermo Fisher Scientific) according to the manufacturer’s instructions. After the cells were transfected with pTurbo-Cre the cultures were protected from light using aluminum foil shielded culture dishes and a microscope (Zeiss Axiovert 200M) with blue-light filter excluding light with λ: 380–500 nm.

### FACS Analysis and Sorting of ChR2-eYFP Positive and Negative RGl Cells

On the 3rd post-transfection day, enhanced Yellow Fluorescent Protein (eYFP)-positive and negative cells were separated using fluorescence activated cell sorting. About 1 - 1.5 × 10^6^ cells were harvested by trypsinization and collected in 2 ml “sorting buffer” (1 mM EDTA and 0.4% bovine serum albumin in PBS). Aliquots of the cell suspensions were introduced into the FACS instrument (BD FACSAria II; BD Biosciences) in order to check the intactness of the cell preparation and to set the optimum parameters for separating eYFP positive and negative cells (FSC and SSC analyses; gate settings; see Supplementary Material S2). Using “high” flow rate (about 65 μl/min; flow grade 10), about 3 % of intact single cells showed high fluorescence and about 78% no fluorescence at all in FITC (fluorescein isothyocyanate) channel. Highly fluorescent and non-fluorescent cells were collected and cultivated separately.

### Differentiation of Radial Glia-Like Neural Stem Cells

Neuronal differentiation of radial glia-like neural stem cells were induced as described before (Markó et al., 2011). Briefly, EGF was withdrawn from the medium of confluent cultures of RGl cells. Confluent cultures were washed with DMEM/F12 (1/1) medium (Sigma) containing 1% B27 (Gibco), and were grown further in this medium without EGF supplementation. Half of the medium was changed every second day.

### Immunocytochemistry

Radial glia-like cells or RGl-derived neuronal cultures were fixed with 4% (w/v in PBS) paraformaldehyde (Taab) for 20 min at room temperature, then permeabilized with 0.1% Triton-X 100 (Sigma) for 5 min. For blocking non-specific antibody binding, the fixed, permeabilized cultures were incubated with 2% bovine serum albumin (Sigma) in PBS (BSA-PBS) for 1 h at room temperature. First-layer antibodies (**Table 1**) were diluted in BSA-PBS. Fixed cells were incubated with the primary antibodies at 4°C, overnight.

**Table 1:** Primary antibodies used.

Antibody	Immunogen	Source	Dilution
Anti- RC2 mouse IgM monoclonal	E14 - E17 rat fetal brain	Developmental Studies Hybridoma Bank; University of Iowa, United States	1/500
Anti-nestin mouse IgG monoclonal	A recombinant 150 amino acid fragment from human Nestin,	Abcam; Cambridge, United Kingdom	1/1000
Anti-GFAP rabbit IgG polyclonal	GFAP from cow spinal cord	DakoCytomation Denmark Glostrup	1/1000
Anti-βIII-tubulin; mouse IgG monoclonal	Carboxyl-terminal sequence of human β-tubulin isotype III	Sigma–Aldrich; St. Louis, MO, United States	1/500
Anti-Pax6; mouse IgG monoclonal	Recombinant chick PAX6 a.a 1-223 protein made in E. coli	Developmental Studies Hybridoma Bank; University of Iowa, United States	1/500
Anti-Sox2; rabbit Ig polyclonal	N-terminal (1-100) region of human SOX2 protein	Abcam; Cambridge, United Kingdom	1/250

After washing with PBS, the cultures were incubated at room temperature for 1 h with fluorochrome-conjugated secondary antibodies: anti-mouse-Alexa-594 and anti-rabbit-Alexa-488 (Invitrogen) diluted in PBS to 1/1000. After washing, the preparations were mounted with Mowiol (Calbiochem) containing 10 μg/ml bisbenzimide (Hoechst 33258; Sigma). Immunocytochemical staining was analyzed with a Zeiss Axiovert 200M inverted fluorescent microscope.

### Microscopic Control of Expression of the ChR2-eYFP Fused Protein

Using the AxioVision 4.8 (Zeiss) program, cells were outlined and the area and fluorescence-intensity of the outlined spots were measured. Fluorescence intensity values were related to 1 μm^2^ cell areas. Averages and standard deviations were calculated from data of 16–25 randomly selected non-induced and 5-day-induced cells (see Supplementary Material S3).

### Electrophysiological Recordings

*In vitro* whole-cell patch clamp recordings were performed from ChR2 expressing or ChR2 non-expressing RGl and RGl-derived neuronal cultures grown on glass coverslips. Cells were visualized with the aid of DIC (differential interference contrast microscopy) optics and EYFP expressing cells were visualized with the Andor Spinning Disk Confocal system (Andor Technology Ltd) mounted on a Nikon FN1 upright microscope. During recordings coverslips were placed into a submerged type recording chamber and perfused (∼2 ml/min, 35–36°C) with a solution containing the following constituents (in mM): NaCl 145, KCl 3, CaCl_2_ 2, MgCl_2_ 1, D-glucose 10, HEPES 10, osmolality 300 mmol/kg, and bubbled with carbogen (95% O_2_, 5% CO_2_). 4–10 MΩ recording pipettes were fabricated from borosilicate glass capillaries (1.5 mm O.D.) with a P1000 puller (Sutter Instruments) and filled with intrapipette solution containing (in mM): KCl 130.0, CaCl_2_ 0.5, MgCl_2_ 2.0, EGTA 5.0, HEPES 10.0. (pH 7.2 with KOH). For membrane current recording, cells were voltage clamped at -65 mV membrane potential with an MultiClamp 700B Amplifier (Molecular Devices) and were stimulated with 50 ms blue light laser pulses (λ: 488 ± 20 nm), gradually increasing the laser power. Resting states and responses to the stimulation were recorded with pClamp 10.3 software (Molecular Devices) and the data were offline analyzed with Clampfit 10.3 software.

### Time-Lapse Microscopy

For video microscopy, non-induced and neuronally differentiated RGl cells were grown in 35 mm Petri dishes with special optical bottom (high glass bottom μ-Dish; Ibidi). Cultures were kept at permanent 37°C temperature and in 5% CO_2_ and 95% air atmosphere within a mobile mini-incubator (Szabó et al., 2011) attached to the microscope stage. Time-lapse recordings were performed with Zeiss Axiovert 200M inverted fluorescence microscope. For light stimulation and fluorescence images, an epifluorescence filter set (band-pass filters for excitation λ: 470 ± 40 nm and for emission λ: 525 ± 50 nm) was used. The cells were stimulated with light for 300 ms in every 5 min with a light intensity of 0.13 mW/ mm^2^. Images were acquired with a 10x objective at the end of each illumination period (in every 5 min) for 12 h, both in phase contrast and epifluorescence optical modes. Light-stimulation and video microscopy was started exactly 24 h and 120 h after the withdrawal of EGF (e.g., the onset of induction) in case of 1-day and 5-day induced populations, respectively.

The effects of light stimulation on cell move were investigated in cultures of non-induced RGl cells, and on 1-day and 5-day induced cells, e.g., on the zero, on the 1st and on 5th days of neuronal differentiation induced by EGF withdrawal (**Table 2**).

**Table 2:** Cells included in motility assays.

Stage of differentiation	Cell populations/experimental groups		
	ChR2-expressing	ChR2-non-expressing	ChR2-expressing;
		
	Illuminated		Not illuminated
Non-induced RGl	*n* = 60	*n* = 60	–
1st day of neuronal induction	*n* = 60	*n* = 60	–
5th day of neuronal induction	*n* = 60	*n* = 60	*n* = 20

At the end of the experiments, cells were fixed with 4% (w/v in PBS) paraformaldehyde (Taab) for 20 min at room temperature and stored in PBS at 4°C for immunocytochemical analysis.

### Analysis of Cell Motility

Time-lapse images were opened in WTrack program (Gönci et al., 2010) and 20 cells were selected in every starting image for movement tracking. As selection criteria, the tracked cells should not leave the microscopic field during the 12-h recording period. A not moving reference point was also selected on each microscopic field in order to correct artifacts due to potential shifts of the frame. The movements of each selected cell were tracked by clicking on the center of the cell and the coordinates were recorded from image to image by WTrack program. The cell coordinates were corrected with the changes in the coordinates of the reference point. The real displacement was determined according to the magnification and the image resolution: 1 pixel corresponded to 0.645 micrometer. Using the corrected cell coordinates, the distances made by cell centers from frame to frame were calculated according to the two dimensional Euclidean distance

$$d(p_{i},p_{i+1})=\sqrt{{\left(x_{i+1}-x_{i}\right)}^{2}+{\left(y_{i+1}-y_{i}\right)}^{2}}*0.645$$

where p_i_ and p_i+1_ are the positions in the actual moment and 5 min later, respectively and 0.645 coefficient was used to calculate distances (d) in micrometer. The resulting data showed the displacement in μm/5 min. Summarizing the frame-to-frame distances

$$d_{\mathrm{total}}=\overset{N-1}{\underset{i-1}{\Sigma }}\sqrt{{\left(x_{i+1}-x_{i}\right)}^{2}+{\left(y_{i+1}-y_{i}\right)}^{2}}*0.645$$

the total distance of cell displacement in 12 h was determined for each tracked cell (*n* = 60 for each differentiation stage, and *n* = 20 for ChR2-expressing but not illuminated cells).

### Statistics

Statistical analyses were performed with R statistic programming (R Core Team, 2016). The graphs were plotted with ggplot2 package. In each case *p* < 0.05 (^∗^), *p* < 0.01 (^∗∗^), and *p* < 0.001 (^∗∗∗^) were considered statistically significant.

The μm values of total distances made by each cell during 12 h (**Figure 5**) were categorized as smaller than 200 μm, between 200 and 400 μm and longer than 400 μm. The data were plotted and statistical significance was determined with Pearson’s Chi-squared test in each contingency table, then paired wise comparisons were performed by *post hoc* Fisher test, where *p*-values were adjusted with Bonferroni method. The electrophysiological responses to illumination (**Figure 4**) and the total distance data (**Figure 6**) were plotted as boxplots and the significances were determined with Wilcoxon–Mann–Whitney test. The significances in the distribution of total distances made by 5-day induced ChR2+ and ChR2- cells in 12 h with or without illumination (**Figure 7**) were determined by Kruskal–Wallis rank-sum test and Dunn’s test.

## Results

### Channelrhodopsin Expressing Radial Glia-Like Neural Stem Cells

**FIGURE 1:**
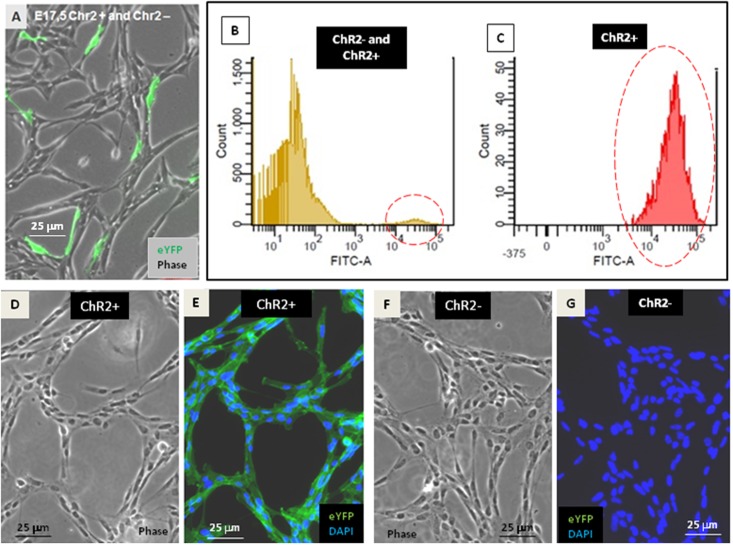
Establishment of ChR2-expressing and non-expressing cell populations. After transfection with pTurbo-Cre plasmid, less than 5% of RGl-cells expressed the ChR2-eYFP fused protein as it was shown by fluorescent microscopy **(A)** and by cytometric analysis (**B,C**; see also Supplementary Material S2). FACS-separated ChR2-eYFP expressing (ChR2+; **D,E**) and ChR2-eYFP non-expressing (ChR2-; **F**,**G**) cells grown in separate cultures displayed similar cell shape.

*In vitro* neurogenesis by optogenetically modified radial glia-like (RGl) neural stem cells (Markó et al., 2011) (see Supplementary Material S1) provided the model for studying the motility responses of differentiating neural cells to ionic stimulation and to compare the effects of stimulation in different stages of RGl cell differentiation. For the presented studies, RGl cells were prepared from the forebrain of channelrhodopsin expressing transgenic (Madisen et al., 2012) mouse embryos on the 17.5 post-conception day. RGl cells carrying the floxed construct for channelrhodopsin-eYFP were grown in AK-c[RGDfC]-coated dishes in serum-free conditions. The cells displayed spindle-like epithelial morphology and radial glia-like immunmarkers (Markó et al., 2011) (Supplementary Material S1) similarly to wild-type RGl cells. When reaching confluency, the cells were transfected with pTurbo-Cre plasmid (Hug et al., 1996) and were let to grow for another week, when about 5% of RGl cells showed eYFP-fluorescence (**Figure 1A**). The cells were collected by trypsinization and eYFP-ChR2-expressing cells were separated from non-expressing cells by FACS (see Supplementary Material S2). eYFP-ChR2-positive (**Figure 1B**) and negative (**Figure 1C**) cells were propagated in separate cultures and were characterized further (**Figures 1D–G**).

**FIGURE 2:**
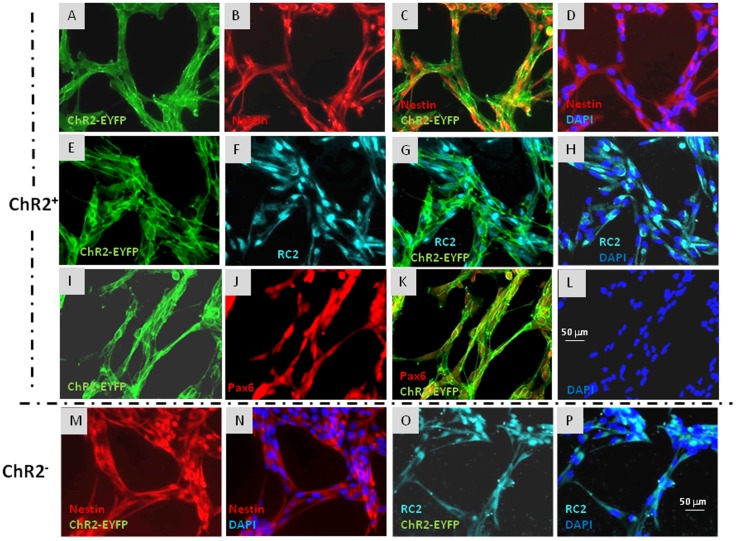
Immunocytochemical characterization of ChR2-eYFP expressing and non-expressing RGl cells. ChR2-eYFP expressing RGl cells were identified by eYFP fluorescence **(A,C,E,G,I,K:** green**).** Both ChR2+ **(A–L)** and ChR2- cells **(M–P)** displayed radial glial markers as nestin (**B–D,M,N**: red), RC2 (**F–H,O,P**: greenish blue) and Pax6 (**J,K**: red). Blue color shows DAPI staining of cell nuclei. All pictures were taken at the same magnification. Scale bars are presented on **(L,P)**.

ChR2-expressing cells were protected from light by growing in aluminum foil covered dishes, fed in a semi-dark sterile hood and examined with blue light filter in daily microscopic investigations. Under these conditions, morphological and immunohistochemical differences were not revealed between ChR2-expressing and non-expressing cells (**Figure 2**).

**FIGURE 3:**
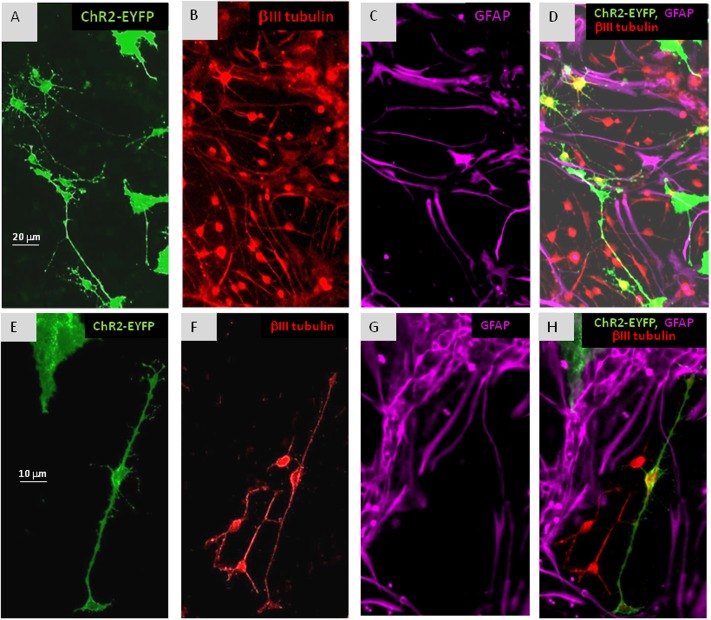
In cultures of mixed ChR2+ and ChR2- cells, withdrawal of EGF resulted in the appearance of βIII-tubulin immunopositive (**B,D,F,H:** red) neuronal precursors among both, ChR2+ (eYFP fluorescent; **A,D,E,H:** green) and ChR2- cells in 5 days. ChR2+ was expressed by GFAP-immunoreactive (**C,D,G,H:** purple) and GFAP negative substrate attached cells, as well. The process-bearing shape of βIII-tubulin immunopositive neuronal precursors is demonstrated in higher magnification on **(E–H)**.

Both ChR2-expressing (**Figures 2A–L**) and non-expressing cells (**Figures 2M–P**) displayed nestin, RC2 and Pax6 immunoreactivity. To compare the neurogenic capacity of ChR2-expressing and non-expressing RGl cells, mixed (non FACS-separated) cultures containing both ChR2-expressing and non-expressing cells were induced by withdrawal of EGF from the medium. Induction of neuronal differentiation resulted in cell aggregation, and a number of cells died regardless of ChR2 expression as it was found previously in induced cultures of wild-type RGl cells (Markó et al., 2011). βIII-tubulin-positive neuronal precursors appeared among both, eYFP-positive ChR2-expressing and eYFP- negative (ChR2-non-expressing) cells by the 5th day of induction (**Figure 3**) indicating that under the same culturing and induction conditions, both ChR2-expressing and non-expressing cells could generate neurons. In comparison either to “wild-type” (Markó et al., 2011) or to ChR2 construct carrying but not expressing RGl cells, no changes were revealed in the morphological or immunohistochemical features of ChR2 expressing RGl cells. The ChR2 expression did not prevent the neuron production by RGl cells. The effect of illumination and all motility studies were carried out on cultures of FACS-separated cells which either expressed the ChR2-eYFP construct, or were ChR2-negative according to the absence of detectable eYFP fluorescence. The intensity of eYFP fluorescence measured in non-induced RGl stem cells and in RGl-derived neurons (Supplementary Material S3) indicated that the expression was not lost in the course of neuronal induction.

### Photostimulation and Video-Imaging of RGl Cells

Illumination with blue (λ: 488 nm) laser light was used to open light-gated channelrhodopsin cation-channels (Zhang et al., 2010) and thus, to modify the ion distribution in non-induced RGl stem cells, and in differentiating progenies on the 1st and 5th days after EGF withdrawal.

**FIGURE 4:**
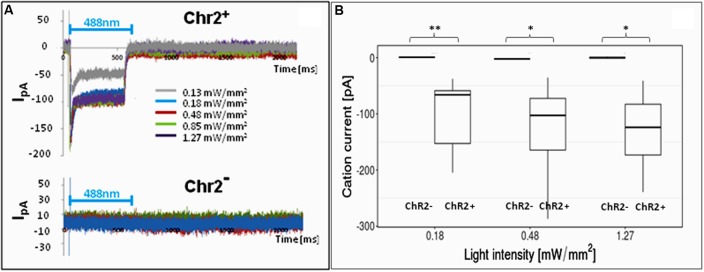
Electrophysiological responses to illumination. In response to illumination ChR2-expressing RGl cells displayed inward cation current [I in picoampers (pA)] depending on the laser intensity. ChR2-non-expressing cells did not respond with ion currents to light stimulation. **(A)** A Representative series of recordings from ChR2+ (upper graph) and ChR2- RGl cells. **(B)** distribution of peak ion currents in response to illumination with different light intensities (*n* = 4 for ChR2-, and *n* = 7 for ChR2+ cells).

In order to find the right parameters for light stimulation, the appropriate intensity of laser illumination was determined by patch-clamp assays (**Figure 4**) and by checking the survival of cells illuminated in every 5th minutes for 12 h. Repeated illumination at an intensity equal or higher than 0.25 mW/mm^2^ caused important cell decay by the end of the 12-h exposure (Supplementary Material S4), while light stimulation below 0.2 mW/mm^2^ did not cause cell damages and did not impair neuronal differentiation.

For 12-h-long motility assays, the cells were repeatedly illuminated with blue light with 0.13 mW/mm^2^ light intensity. This intensity elicited measurable inward cation currents (**Figure 4**), and did not cause decay of either non-induced RGl cells or RGl-derived neuronal precursors. Cultured cells in optical culture dishes were placed in a mini-incubator stuck to the microscope table and were illuminated for 300 ms in every 5 min. Images in both phase contrast and epifluorescence optical modes were taken at the end of each 300 ms illumination period.

### Analysis of Cell Displacement

The centers of individual cells were tracked on consecutive frames of the time-lapse video images and the cell positions were determined using the WTrack program (Gönci et al., 2010) (Supplementary Material S5). The *x, y* coordinates of individual cell positions were related to a non-moving reference point and the move of labeled cell centers were calculated from frame to frame. The trajectories of individual cells were plotted (Supplementary Material S5) and the total distances made by cell centers were calculated by summarizing the lengths of 5-min displacements, starting from the position on the first image (*t*_0_) and ending with the positions at the end of the 12th hour (*t*_143_). The displacement of the cell center was accepted as migration if the tracked cell center moved twice as far as the longest dimension of the cell during the 12-h observation period. As the average length of RGl cells did not exceed 100 μm, displacement was accepted as migration if the cell center moved as far as 200 μm as a minimum in 12 h.

The move of ChR2-expressing and non-expressing (control) cells was analyzed in non-induced, 1-day induced, and 5-day induced populations (**Table 2**).

**FIGURE 5:**
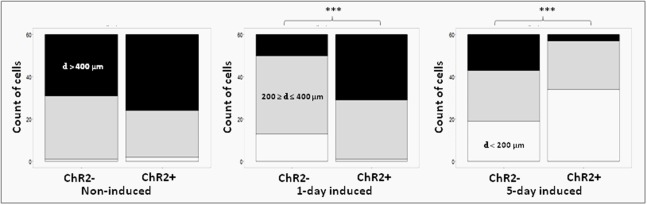
Proportions of cells with different displacement activities. The move of ChR2+ and ChR2- cells was analyzed in non-induced (n_ChR2+_ = 60; n_ChR2-_ = 60), 1-day induced (n_ChR2+_ = 60; n_ChR2-_ = 60) and 5-day induced (n_ChR2+_ = 60; n_ChR2-_ = 60) cultures in three independent experiments. The number of cells which moved to defined distances during the 12-h investigation period (black: d > 400 μm; gray: = 200 μm ≥ d ≤ 400 μm; white: d < 200 μm) was determined. Differences in cell displacements were calculated for ChR2+ and ChR2- cells in each developmental stages. Statistical significances were determined with Pearson’s Chi-squared test and post-hoc Fisher test. The significant (*p* < 0.001) alterations are indicated by ^∗∗∗^.

**FIGURE 6:**
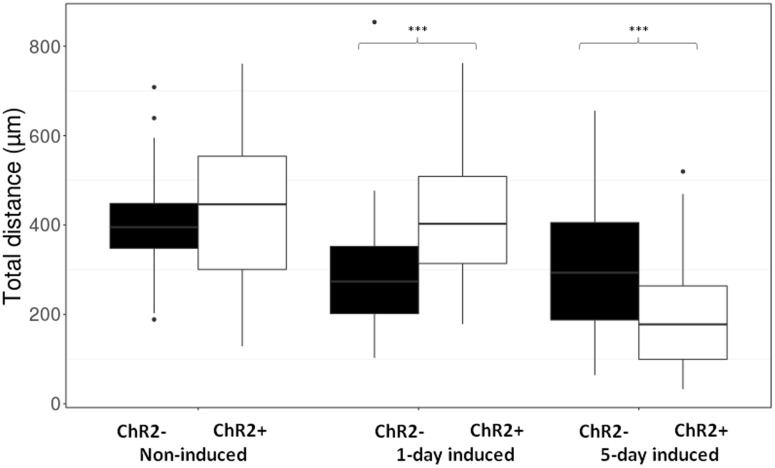
The length of routes. Boxplot presentation of the distribution of total distances made by cells during the 12-h period of repeated illumination in different (non-induced, 1-day induced and 5-day induced) stages of differentiation. Significances were determined by with Wilcoxon–Mann–Whitney test. (*n* = 60 in each experimental group).

**FIGURE 7:**
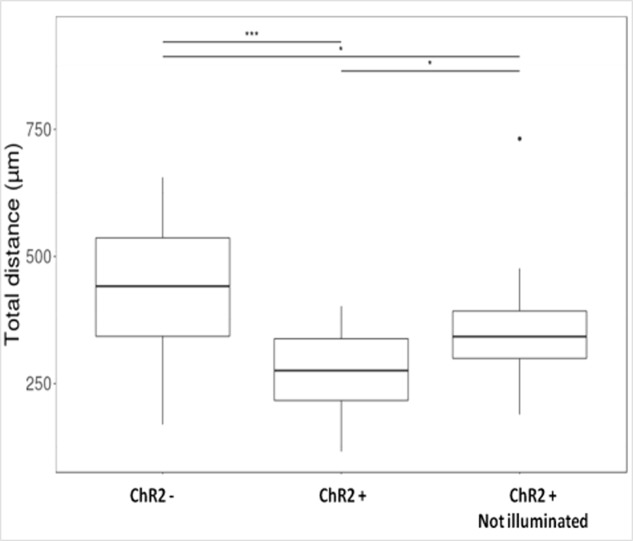
Boxplot presentation of the total distances made by cells on the 5th day of induction. Motility of ChR2+ and ChR2- (n_ChR2+_ = 20; n_ChR2-_ = 20) cells during the 12-h period of repeated illumination or that of ChR2+ cells (n_ChR2+_ = 20) without illumination were analyzed. Significances were determined by Kruskal–Wallis rank-sum test and Dunn’s test.

Displacement data obtained on illuminated ChR2-expressing and non-expressing (control) cells demonstrated that cell displacement decreased with the advancement of neuronal differentiation in both ChR2-expressing and non-expressing cells (**Figures 5**, **6**). The number of cells migrating longer than 400 μm in 12 h (**Figure 5**) and the median of displacement distances (**Figure 6**) decreased by the 5th day of induction. In ChR2-negative populations, a mild reduction of motility was detected as soon as 24 h after the onset of induction, and the displacement activity of process-bearing cells with neuron-specific tubulin immunoreactivity was markedly reduced on the 5th day of induction. The light-sensitive cells, however, displayed different displacement-activity if exposed to repeated light stimulation. Illumination slightly (but not significantly) increased the displacement activity of non-induced ChR2-expressing RGl stem cells (**Figures 5**, **6**). On the first day of neuronal induction, the number of ChR2-expressing cells with longer migratory routes increased further (**Figure 5**) and the average distance of cell displacement was significantly higher than that in ChR2- populations (**Figure 6**). On the 5_th_ day of induction, however, light stimulation markedly reduced the displacement activity: the migratory routes made by illuminated ChR2-expressing cells were significantly shorter than those of ChR2 non-expressing or ChR2-expressing but not illuminated cells (**Figure 7**).

The data clearly demonstrated that light-induced inward cation fluxes modified the motility of developing neural cells on a differentiation stage-dependent way.

The migration activity of 5-day induced ChR2-expressing cells decreased even without any direct exposure to blue light (**Figure 7**) in comparison to ChR2-non-expressing cells. In response to blue light illumination, however, the displacement activity of ChR2-expressing cells, was more significantly reduced. The data indicate that physiologically effective amount of ions could move through ChR2 channels even in the absence of targeted illumination with blue light. Stimulation with blue light, however, caused significantly enhanced inward cation currents and modified significantly the displacement activity of the cells.

## Discussion

The insertion of ChR2-eYFP and Cre-recombinase coding gene constructs and the expression of the corresponding proteins did not cause important changes in morphological features of RGl cells. Motility assays, however, verified that light-triggered cation influxes modified the displacement activity of the cells on a developmental stage-dependent way.

For evaluating cell motility, several difficulties were faced. The characteristic elongated shape of RGl cells forced us to track the “cell centers” determined by the widest and tallest cell dimensions, which necessarily corresponded to the location of the cell nucleus (verified also with DAPI staining). Intracellular displacement of nuclei is a well-known phenomenon (Chang et al., 2015), which is evident in migratory cells but does not necessarily mean cell-displacement in cells with elongated shape (Tsai and Gleeson, 2005). Nuclear motility was demonstrated on isolated RGl cells as well (Szabó et al., 2011). To distinguish cell displacement from nucleokinesis, the move of the cell center was compared to the longest dimension of RGl cells. Migration was voluntarily defined: the displacement of the cell center was accepted as migration if the tracked cell center moved twice as far as the longest dimension of the cell, during the 12-h observation period. As the average length of the longest dimension of RGl cells did not exceed 100 μm, cells were regarded migrating if the center moved for minimum 200 μm in 12 h.

As an other difficulty, the rate of the transgene expression may change with cellular states and differentiation. Differentiation-dependent alterations in the expression of light-gated cation channels might result in apparent variations of light-evoked ion fluxes and thus, might lead to a misinterpretation of ion distribution-caused motility changes. To overcome this uncertainty, the expression of light-gated ChR2 cation channels was assessed by measuring the intensity of eYFP fluorescence in non-induced RGl stem cells and RGl-derived neurons. Data showed that the fluorescence intensity of cells did not change significantly with differentiation. The data allowed presuming that the density of ChR2 molecules was similar in RGl stem cells and in differentiating progenies.

Based on 12-h displacement distances, the majority of non-induced RGl stem cells proved to be “migratory,” with a moving distance exceeding 200 μm, and the migration activity was not modified significantly by light-induced cation influx. The observation indicated that inherent features of non-induced RGl cells support motility, and opening the light-gated cation channels could not add important spur to motion.

As it was expected, ionic stimulation evoked different responses in cells induced for neuronal differentiation. On the first day of induction, RGl cells show elongated shape without fine processes, and are arranged in string-like patterns by lining along each other. They express proneural genes including *ngn2* and *mash1*, but do not display neuronal characteristics: they do not express *math2* or neurotransmitter-specifying genes (Supplementary Material S1), and do not show immunopositivity for βIII-tubulin or vesicular neurotransmitter transporters (Markó et al., 2011). In this “progenitor” stage, the motility decreased in ChR2- control cells, while opening of light-gated cation channels increased the motility in 1 day induced ChR2+ RGl cells. The finding indicates that environmental stimuli which can influence the intracellular ion distribution can alter the migratory activity of radial glia-like progenitors after the onset of differentiation.

By the 5th day of induction, RGl cells start to display neuronal characteristics (Markó et al., 2011) (Supplementary Material S1). This stage of differentiation does not represent a final maturation-state of RGl-derived neurons, and has been regarded as a neuronal precursor stage. It is well known that *in vivo*, the outgrowth and elongation of neuronal processes are delayed until the neuronal precursor cell has settled at their tissue destination, and mature neurons do not migrate (Chang et al., 2015). As decreasing migration activity indicates the stage of maturation of neuronal precursors, this “precursor stage” was chosen for motility studies. In the “precursor” stage, the displacement activity of RGl-derived cells decreased regardless of light stimulation. In response to repeated illumination, however, the motility of ChR2+ precursor populations was markedly reduced in comparison to ChR2-non-expressing cells. The reduced migration activity may indicate some acceleration of neuronal maturation in response to repeated ionic stimuli. Reduced displacement activity of 5-day induced ChR2-expressing cells, however, was evident without targeted illumination. The finding pointed toward the possible occurrence of ion fluxes through ChR2-channels opened by scattered light which could not be completely prevented during cell handling and video imaging. Non-intended light-stimulation of ChR2+ cells could easily modify the intracellular ion distribution, and so, could interfere with cell motility and other cell physiological/differentiation processes. In comparison to targeted stimulation with blue light, however, the motility of non-illuminated ChR2+ cells was significantly less modified.

Previous studies (Markó et al., 2011) demonstrated that RGl cells display voltage-dependent cation channels, while the channel composition and the ionic responses to electric stimuli change with the advancement of neuronal differentiation: RGl-derived neurons were shown to produce rapid inward Na^+^-currents in response to electric stimulation from the 6–7th day of induction. While ChR2-expressing RGl cells responded to illumination with inward cation fluxes in all stages of differentiation, the applied stimulation did not evoke propagating action potentials in the 5-day induced RGl-derived neuronal precursors and the light intensity needed for saturation of the steady-state photocurrent was low in comparison to that shown in cultures of hippocampal pyramidal cells (∼1 mW/mm^2^; Mattis et al., 2011). The different types of neurons in the B6;129S-Gt(ROSA)26 Sor^tm32(CAG-COP4^∗^H134R/EY FP)Hze^/J mouse strain, however, may require individual conditions for photo activation (Madisen et al., 2012). In our case, even the most developed (5-day induced) cells can not be regarded fully matured excitable cells, and are not expected to exhibit proper neuron-specific machinery for rapid redistribution of ions (Markó et al., 2011). In case of neural stem/progenitor cells, the saturation intensity should be rather compared to that of non-excitable cells: In ChRH134 transfected HEK cells, the theoretical EC50 (at 450 nm) of steady-state response was calculated to be bellow of 1 mW/mm^2^ (Lin, 2011). As the saturation density varies with the type of cells and with the actual cell physiological characteristics, the low light intensity for saturating the steady-state photocurrent was accepted as a characteristics of RGl cells derived from the B6;129S-Gt(ROSA)26 Sor^tm32(CAG-COP4^∗^H134R/EY FP)Hze^/J mice.

In the developing brain, stem/progenitor and neuronal precursor cells are exposed to stimuli which can influence the distribution of ions but do not cause large depolarizations and not necessarily provide over-threshold stimuli for generating spike potentials in maturing neurons. These influences, however, can affect basic cell biological and developmental processes. Similarly to the studies of Stroh and co-workers on mouse embryonic stem cells (Stroh et al., 2011), our data suggest that optogenetical ionic stimulation can modify the processes underlying neuronal differentiation. By altering the intra/extracellular ion distribution, optogenetic stimulation may be used also for *in vivo* tuning the motility of neural stem- and progenitor cells. Accelerating or slowing down the move of neural progenitors may interfere with the highly orchestrated processes of neural tissue genesis and may accelerate the understanding on conditions needed for the integration of novel neurons in the developing or regenerating neural tissue.

## Ethics Statement

This study was carried out in accordance with European (2010/63/ EU Directives) and Hungarian (40/2013) legislations. The protocol was approved by he National Food-chain Safety Office of Hungarian Government under the licence 22.1/354/3/2011.

## Author Contributions

Study concept and design: EM, KM, and TK. Acquisition of data: TK, NP, TA, AJ, and ZK. Analysis and interpretation of data: TK, AJ, NP, and ZK. Drafting of the manuscript: TK and EM. Critical revision of the manuscript for important intellectual content: EM, KM, AJ, and ZK. Statistical analysis: AJ and NP. Obtained funding: EM and TK. Administrative, technical, and material support: TA and ZK. Study supervision: EM, TA, and AJ.

## Conflict of Interest Statement

The authors declare that the research was conducted in the absence of any commercial or financial relationships that could be construed as a potential conflict of interest.
