# Supplementary material for: Differentiation-Dependent Motility-Responses of Developing Neural Progenitors to Optogenetic Stimulation
Source: Front Cell Neurosci. 2017 Dec 19;11:401. doi: 10.3389/fncel.2017.00401 (PMC5742229; doi:10.3389/fncel.2017.00401)
Supplement: Supplementary file 1 [file Presentation_1.pdf]

Supplementary Material

Differentiation-dependent motility-responses of developing neural progenitors to optogenetic stimulation

Tímea KŐHIDI[1], Attila Gy. JÁDY[1,2], Károly MARKÓ[3], Noémi PAPP[1], Tibor ANDRÁSI[5], Zsuzsanna KÖRNYEI[1,6], Emília MADARÁSZ[1*]

*** Correspondence:** Emília Madarász: madarasz.emilia@koki.mta.hu

S1. Brief description of the characterization and in vitro differentiation of radial glia-like (RGl) neural stem cells

Using selective adhesion to AK-c[RGDfC] (Markó et al., 2008) and serum free propagation in the presence of EGF (epidermal growth factor), RGl cells can be isolated and cloned from various parts of the embryonic (E 14-15, E17-18), newborn (P0-3) and adult (P50-75) mouse brains (Markó et al., 2011). RGl cells display similar molecular, morphological and developmental characteristics, regardless of tissue origin (S1 Fig.1)

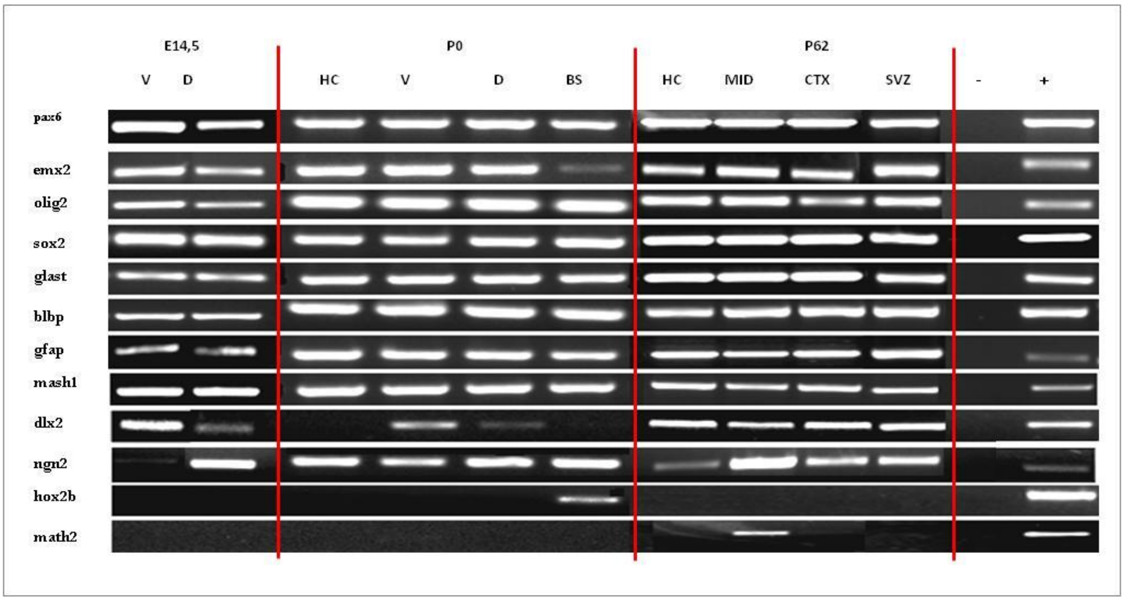

S1 Fig.1. Expression of regional, radial glial marker and proneuronal genes by RGl cells isolated from various regions of E14.5 embryonic, newborn (P0) and adult (P62) mouse brain. V: ventral; D: dorsal; HC: hippocampus; BS: brain stem; MID: midbrain colliculus superior; CTX: cortex; SVZ: forebrain subventricular zone; - and +: negative and positive controls, respectively

PCR primer sequences used for characterization of the gene expression profile of RGl cells

Gene	Sequences
Pax6	Forward: 5'-ACGAAAGAGAGGATGCCTC-3'
	Reverse: 5'-CCCAAGCAAAGATGGAAG-3'
Sox2	Forward: 5'-GCCCTGCAGTACAACTCCAT-3'
	Reverse: 5'-ACCCCTCCCAATTCTCTTGT-3'

Blbp	Forward: 5’-ACCCGAGTTCCTCCAGTTC-3’
	Reverse: 5’-CAAAAGCAAGTTCCCATTCA-3’
Glast	Forward: 5’-TGGGTTTTCATTGGAGGGTTG-3’
	Reverse: 5’-CAGTACGTTGGTGGTGGTTCG-3’
Gfap	Forward: 5’-GACTATCGCCGCCAACTGC-3’
	Reverse: 5’-CGTCCTTGTGCTCCTGCTTC-3’
Math2	Forward: 5’-TGAGAATGGCTTGTCCAGAAGG-3’
	Reverse: 5’-TGGTAGGGTGGGTAGAATGTGG-3’
Oct4	Forward: 5’-GGCGTTCTCTTTGGAAAGGTGTTC-3’
	Reverse: 5’-CTCGAACCACATCCTTCTCT-3’
Nanog	Forward: 5’-GCGCATTTTAGCACCCCACA-3’
	Reverse: 5’-GTTCTAAGTCCTAGGTTTGC-3’
Mash1	Forward: 5’-GTTGGTCAACCTGGGTTTTG-3’
	Reverse: 5’-GTGATTCGGGCTTAGGTTCA-3’
Ngn2	Forward: 5’-AAGAGGACTATGGCGTGTGG-3’
	Reverse: 5’-ATGAAGCAATCCTCCCTCCT-3’
Emx2	Forward: 5’-GTCCCAGCTTTTAAGCTAGA-3’
	Reverse: 5’-CTTTTGCCTTTTGAATTTCGTTC-3’
Dlx2	Forward: 5’-CAGGGTCCTTGGTCTCTTCA-3’
	Reverse: 5’-CTGCTGAGGTCACTGCTACG-3’

RGl cells display elongated epitheloid shape and give rise to neurons, astrocytes and oligodendrocytes (S1 Fig. 2), depending on the applied differentiation protocol. Detailed description of differentiation protocols and immunocytochemical characterization of RGl cells and RGl-derived progenies had been presented in Markó et al., 2011.

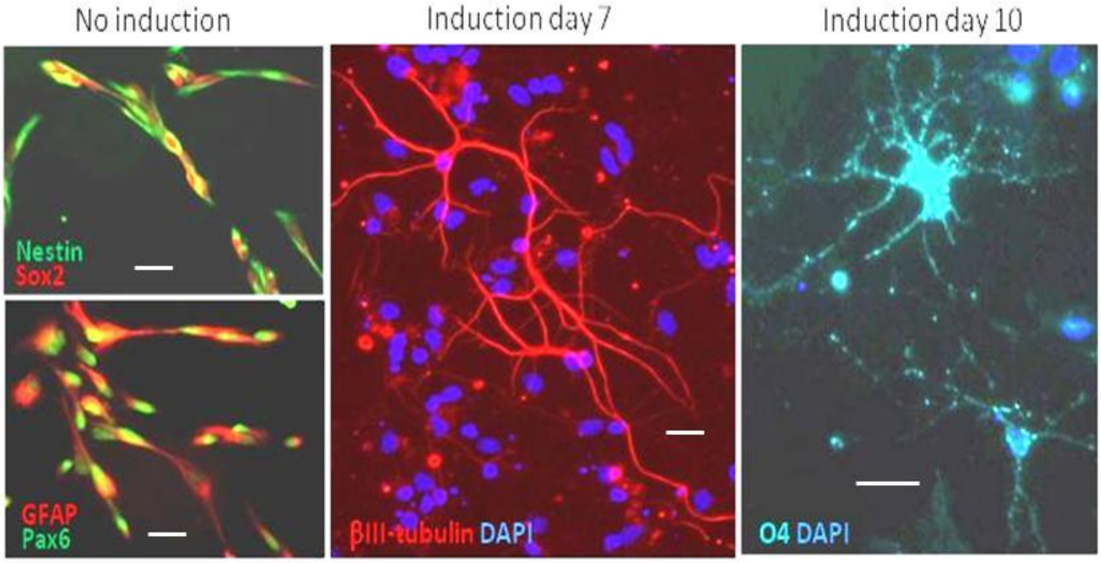

S1 Fig.2. Morphology and immunocytochemical markers of RGl cells and induced progenies. Bars represent 10 μm.

In vitro differentiation proceeds through reproducible steps (S1 Fig.3)

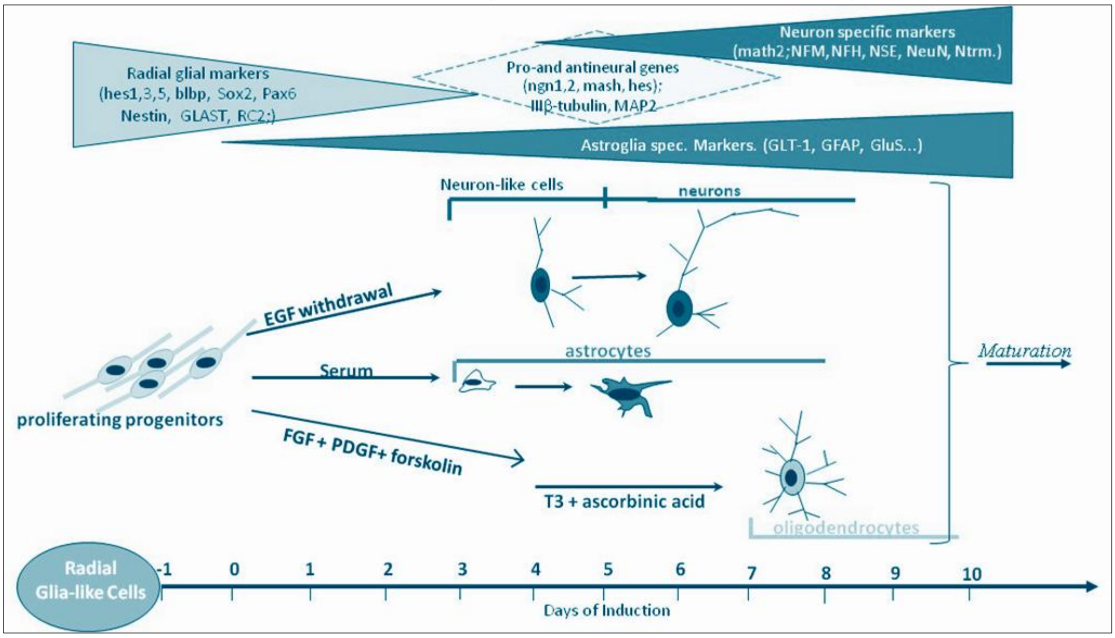

S1 Fig.3. The scheme of in vitro neural differentiation of RGl cells (Modified from Madarasz 2013)

References

1. Markó K, Ligeti M, Mezo G, Mihala N, Kutnyánszky E, Kiss É, et al. A novel synthetic peptide polymer with cyclic RGD motifs supports serum-free attachment of anchorage-dependent cells. Bioconjug Chem. 2008;19(9):1757–66.

2. Markó K, Köhidi T, Hádinger N, Jelitai M, Mezö G, Madarász E. Isolation of radial glia-like neural stem cells from fetal and adult mouse forebrain via selective adhesion to a novel adhesive peptide-conjugate. PLoS One. 2011;6(12). e28538

3. Madarász E. Diversity of Neural Stem/Progenitor Populations: Varieties by Age, Regional Origin and Environment. In Neural Stem Cells - New Perspectives, Dr. Luca Bonfanti (Ed.), ISBN: 978-953-51-1069-9, InTech, 2013. DOI: 10.5772/55678

S2. FACS analysis and sorting of ChR2-eYFP RGl cells

About 1-1.5 x 10^6 cells were harvested by trypsinization and were collected in 2 ml “sorting buffer” (1 mM EDTA and 0.4% bovine serum albumin in PBS). An aliquot of the cell suspension was introduced into the FACS (BD FACSAria II) instrument in order to check the intactness of the cell preparation and to set the optimum parameters for separating eYFP positive and negative cells (S2 Fig.1).

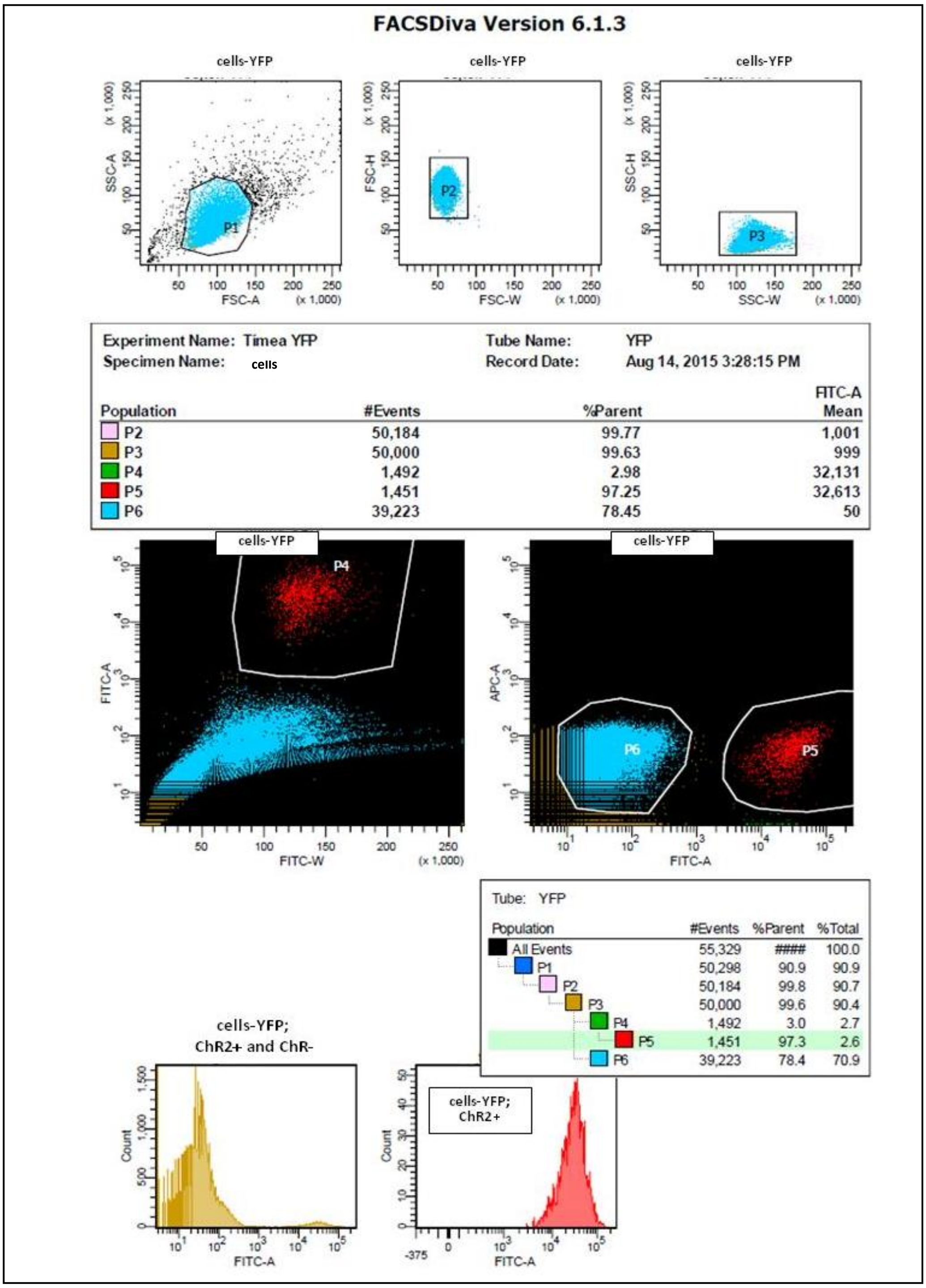

S2 Fig.1

S3. Cell areas measured by AxioVision 4.8 program

Potential changes in expression of the ChR2-eYFP construct was checked by measuring the eYFP fluorescence intensity on images taken on non-induced and 5-day induced populations of RGl cells. Cells were randomly selected and the cell areas and fluorescence intensities were determined by AxioVision 4.8 program (Zeiss, Jena, Germany) (S**3** Fig. 1)

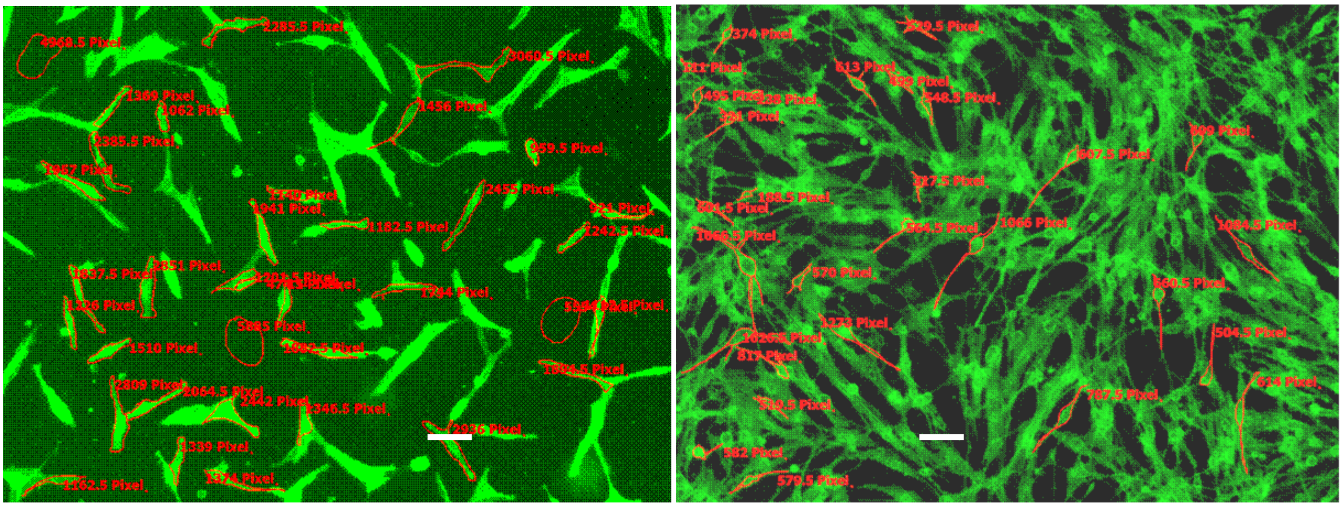

S3 Fig.1. Cell areas and fluorescence intensities of the measured cell areas were measured by AxioVision 4.8 program. Bars represent 20 μm.

The measured fluorescence intensities were divided by the corresponding cell areas resulting in a virtual fluorescence intensity/1 μm^2 (S3 Fig.2).

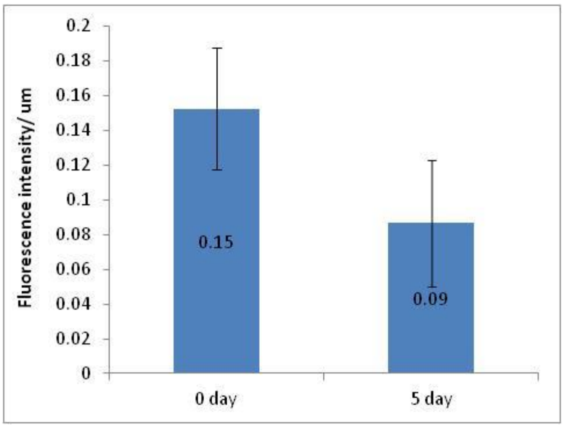

S3 Fig.2. Calculated values of specific fluorescence density of non-induced RGl cells and 5-day induced RGl progenies. Averages and standard deviations of specific fluorescence densities calculated by dividing the fluorescence of each outlined cell shape by the outlined cell area (n>20)

S4. Effect of repeated illumination with different light intensity

Repeated illumination (300 ms duration in every 5 minutes) at an intensity equal or higher than 0.25 mW/mm^2 caused important cell decay in less than 7 hours (S4 Fig.1).

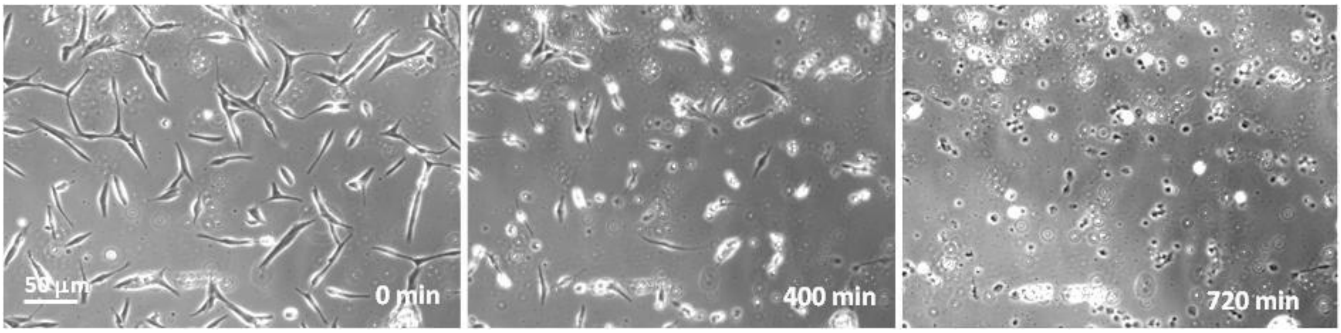

S4 Fig.1. Phase-contrast microscopic images of a RGl cell culture at the beginning (0 min) and during (shown at 400th and 720th min) repeated illumination with 300 ms-long, 0.27 mW/mm^2 blue (λ = 488 nm) laser light impulses in every 5 minutes.

Repeated illumination at a light intensity of 0.13 mW/mm^2 did not cause important cell decay in 12-hour stimulation period (S4 Fig.2)

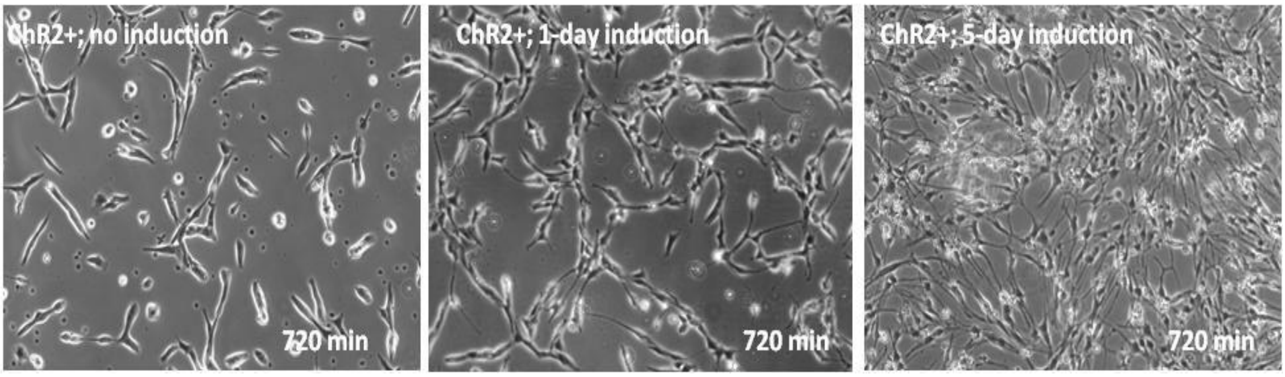

S4 Fig.2. Phase-contrast microscopic images of non-induced, 1-day induced and 5-day induced RGl cell cultures at the end of the 12-hour illumination period.

S5. Determination of cell displacement

The centers of individual cells were tracked on consecutive frames of the time-lapse video images and the cell positions were determined using the WTrack program (Gönczi et al., 2010;). The trajectories of each cell were plotted (S5 Fig.1) and the displacement of cell centers were calculated as it is described in the Materials and Methods chapter.

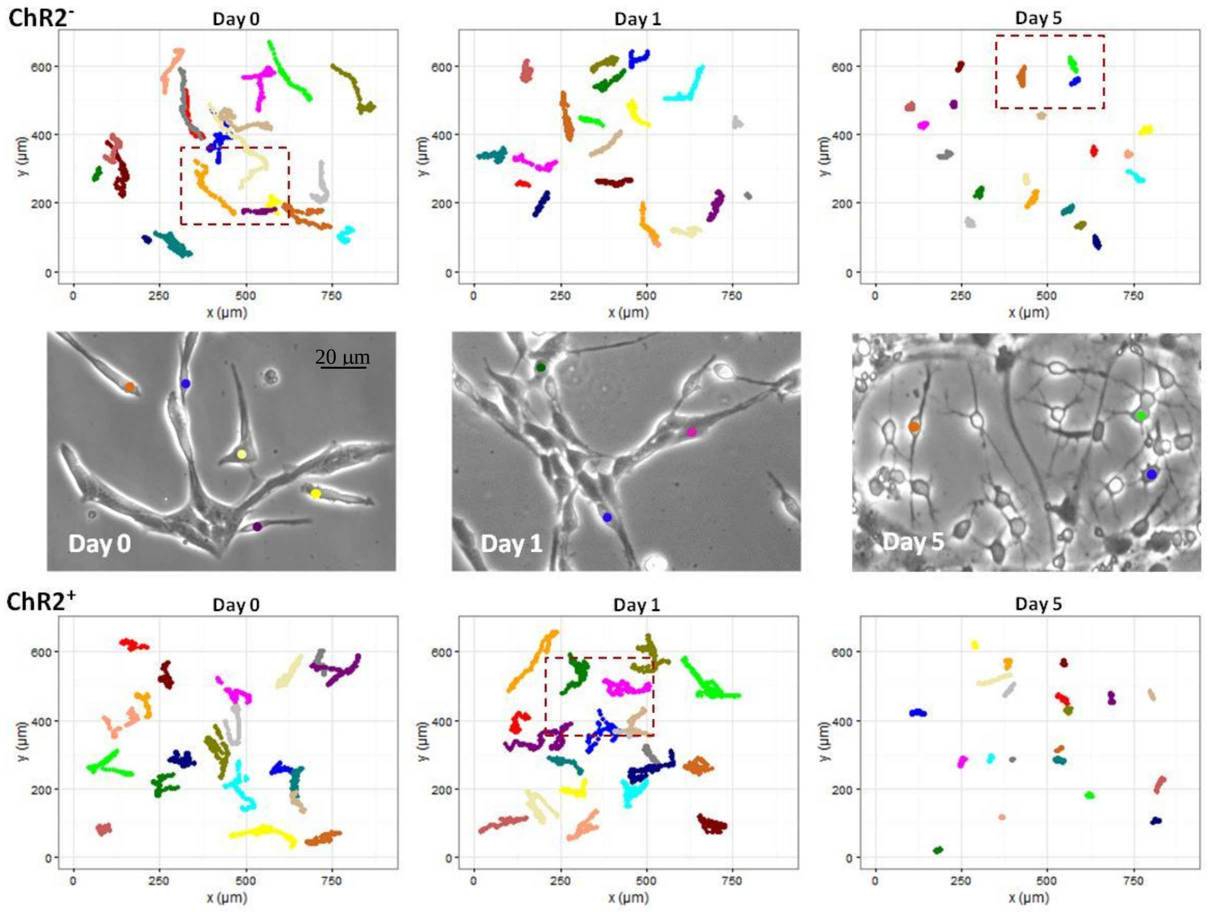

S5 Fig.1. Trajectories of ChR**2**-expressing (ChR**2**+) and non-expressing (ChR**2**-) RGl cells in a representative experiment. Colored spots on phase-contrast pictures show the cell centers, which were tracked on the trajectograms. The pictures were taken at the same magnification. Rectangles indicate the trajectories of cells shown on the pictures.
